# NF-κB Members Left Home: NF-κB-Independent Roles in Cancer

**DOI:** 10.3390/biomedicines5020026

**Published:** 2017-05-25

**Authors:** Carlota Colomer, Laura Marruecos, Anna Vert, Anna Bigas, Lluis Espinosa

**Affiliations:** Stem Cells and Cancer Research Laboratory, CIBERONC. Institut Hospital del Mar Investigacions Mèdiques (IMIM), 08003 Barcelona, Spain; ccolomer@imim.es (C.C.); lmarruecos@imim.es (L.M.); Avert@imim.es (A.V.); abigas@imim.es (A.B.)

**Keywords:** Cancer, NF-κB, Non-conventional pathways, IKKs, IκBs

## Abstract

Nuclear factor-κB (NF-κB) has been long considered a master regulator of inflammation and immune responses. Additionally, aberrant NF-κB signaling has been linked with carcinogenesis in many types of cancer. In recent years, the study of NF-κB members in NF-κB unrelated pathways provided novel attractive targets for cancer therapy, specifically linked to particular pathologic responses. Here we review specific functions of IκB kinase complexes (IKKs) and IκBs, which have distinctly tumor promoting or suppressing activities in cancer. Understanding how these proteins are regulated in a tumor-related context will provide new opportunities for drug development.

## 1. Introduction

Since the discovery of the nuclear factor κB (NF-κB) more than 30 years ago [1] the NF-κB pathway has been the focus of multiple studies owing to its role in the regulation of essential biological processes, such as immune and stress responses, cell survival, or cell maturation. Due to its functional relevance, alterations in NF-κB signaling tend to affect organism homeostasis, leading to tissue damage and, in some cases, to cancer [2]. Thus, gaining insight into the function and regulation of particular NF-κB components is crucial for the future development of effective therapies against a wide variety of diseases that involves NF-κB, including diabetes [3,4], allergies and rheumatoid arthritis [5], Crohn’s disease [6], Alzheimer’s disease [7], or cancer, among others.

The mammalian NF-κB family consists of five transcription factors: p65 (RelA), RelB, c-Rel, p105/p50 (NF-κB1), and p100/p52 (NF-κB2) [8,9,10]. Although RelA, RelB, and c-Rel are synthesized as final proteins, p50 and p52 derive from p105 and p100, respectively, upon proteasomal processing. All of the members can form homo- and heterodimers, and shuttle from the cytoplasm to the nucleus in response to cell stimulation. NF-κB transcription factors are characterized by the presence of a highly-conserved Rel homology domain (RHD) which is responsible for dimerization, DNA binding, and interaction with the inhibitor of κB (IκB) proteins [10]. The IκB proteins, including IκBα, IκBβ, IκBε, IκBγ, IκBζ, Bcl-3, and the precursor Rel proteins p100 and p105, are characterized by the presence of multiple ankyrin repeats, which are protein-protein interaction domains that interact with NF-κB via the RHD [10]. IκBs control the activation of the NF-κB dimers (except for p52-RelB) by masking the nuclear localization signal (NLS) of Rel proteins, thus preventing its nuclear translocation and the subsequent activation of target genes. Therefore, IκB degradation is a tightly-regulated event that is triggered upon a stimulus-response activation of the IκB kinase (IKK) complex. The IKK complex is formed by two catalytic subunits, IKKα and IKKβ, and a regulatory subunit called IKKγ or NF-κB essential modulator (NEMO) [11].

There are a variety of ligands that can trigger the signal transduction resulting in the activation of specific IKK-dependent cascades, being the two principal the classical (or canonical) and the alternative (or non-canonical) NF-κB pathways. In the classical pathway, activated IKKβ by transforming growth factor-β activated kinase 1 (TAK1) is necessary to induce phosphorylation of IκBs on two N-terminal residues (IκBα on Ser32 and Ser36 and IκBβ on Ser19 and Ser23). This event leads to its ubiquitination by the Skp-1/Cul/F box (SCF) family and its proteasomal degradation [11,12]. On the other hand, the alternative pathway depends on the activation of IKKα by the NF-κB inducing kinase (NIK). The IKKα subunit phosphorylates p100 which, under resting conditions, is associated with RelB in the cytoplasm, inducing its processing to p52 [13]. In both pathways, after this processing, the NF-κB transcriptional factors are able to translocate to the nucleus, where they bind to promoter and enhancer regions containing κB sites with the consensus sequence GGGRNNYYCC (N = any base, R = purine, Y = pyrimidine).

As mentioned, NF-κB pathway play an important task in the development and maintenance of cancer, mainly associated with its normal role in inflammation and immune response. However, it is also true that particular NF-κB-related elements can be deregulated in cancer cells, thus exerting less conventional pro- or anti-tumorigenic functions. Examples include the aberrant activity of members of the pathway, genetic aberrations of genes coding for NF-κB family members, autocrine and paracrine production of pro-inflammatory cytokines by the tumor cells, as well as oncogenic activation of upstream signaling molecules. All of these mechanisms lead to altered expression of specific target genes or whole transcriptional programs which, in turn, modify cellular proliferation or apoptosis, tumor-associated angiogenesis, metastasis, or resistance to chemo- and radiotherapy [14,15,16,17,18,19]. In addition, particular members of the NF-kB pathway have been found to exert non-conventional and NF-κB-independent functions that are physiologically relevant, but can also impact some cancer cell capabilities. The present review focuses on the non-conventional functions of the NF-κB pathway family of proteins IKK and IκB that negatively or positively contribute to cancer initiation and progression.

## 2. Breast Cancer

**Figure 1:**
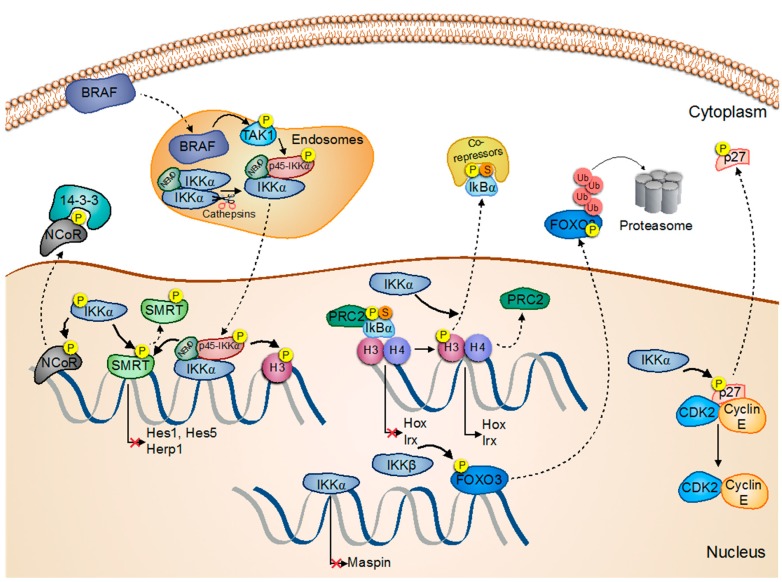
Pro-tumorigenic functions of the NF-κB members. In CRC, IKKα phosphorylates the nuclear co-repressors N-CoR and SMRT, inducing its dissociation from the chromatin. In prostate cancer cells IKKα regulates the gene transcription of the metastasis repressor Maspin. The proteolytic fragment p45-IKKα is activated by BRAF and TAK1 in the endosomal compartment, and upon activation can phosphorylate histone H3 and SMRT. Moreover, nuclear IKKα contributes to the chromatin release of IκBα, and stimulates the nuclear export of p27/Kip1, thereby supporting the proliferation and expansion of tumor cells. On the other hand, IKKβ phosphorylates FOXO3a, leading to its nuclear exclusion and protein degradation. Arrows: 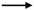 Activation/Regulation/Phosphorylation; 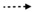 Migration; 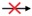 Inactivation.

Both IKKα and IKKβ display oncogenic functions in breast cancer cells that are independent of their role in the NF-κB pathway. In response to estrogen, IKKα increases phosphorylation and recruitment of estrogen receptor alpha (ERα) and steroid receptor coactivator 3 (SRC-3) to estrogen-responsive promoters, including *cyclin D1* and *c-myc*, leading to enhanced gene transcription. Activation of these genes increases estrogen-dependent proliferation of breast cancer cells [20]. IKKα can also cooperate with Notch-1 to induce the transcriptional activation of ERα-dependent genes [21]. On the other hand, IKKα promotes the estrogen-induced transcription of E2F Transcription Factor 1 (E2F1) and facilitates the subsequent activation of several E2F1-responsive genes such as *thymidine kinase 1* (*TK1*), *proliferating cell nuclear antigen* (*PCNA*), *cyclin E*, and *cdc25A*, which are required for cell cycle progression of breast cancer cells [22]. IKKα is also an important contributor to ErbB2-induced oncogenesis, as it supports the expansion of tumor-initiating cells from premalignant ErbB2-expressing mammary glands. Upon activation, IKKα enters into the nucleus of these cells and phosphorylates p27/Kip1 inducing its nuclear export, which results in enhanced cell proliferation [23] (Figure 1).

IKKβ also promotes breast cancer through the phosphorylation of forkhead box O3 (FOXO3a), which triggers its cytoplasmic export and proteasomal degradation, resulting in increased proliferation and tumorigenesis (Figure 1). This mechanism was primarily found in tumors lacking Akt activity since Akt is usually responsible for FOXO3a phosphorylation and degradation [24].

## 3. Prostate Cancer

In prostate cancer, IKKα phosphorylates and activates the mammalian Target of Rapamycin Complex 1 (mTORC1) in phosphatase- and tensin homolog (PTEN)-null prostate cancer cells in a manner dependent on Akt, promoting cell proliferation [25,26]. Similarly, IKKα associates with, and enhances, mTORC2 kinase activity [27]. Of note, it is known that activated Akt promotes cell survival, cell growth and proliferation, and energy metabolism in prostate cancer [28]. IKKα can also phosphorylate the nuclear co-repressor silencing mediator for retinoid and thyroid receptors (SMRT), thus inducing its dissociation from the chromatin and its nuclear export mediated by 14-3-3. This event is a prerequisite for the recruitment of NF-κB to specific promoters such as the cellular inhibitor of apoptosis 2 (cIAP-2) and interleukin 8 (IL-8), leading to increased cell survival [29]. In castration-resistant tumors, nuclear active IKKα represses the transcription of the metastasis-suppressor gene *Maspin* (Figure 1). Accordingly, accumulation of nuclear active IKKα in human and mouse prostate tumors correlates with metastatic progression, reduced *Maspin* expression, and infiltration of receptor activator of nuclear factor κ-B ligand (RANKL)-expressing inflammatory cells [30]. A similar association between IKKα nuclear localization, *Maspin* levels, and cell migration or metastasis has been shown in squamous cell carcinoma cells (see details in Section 5).

## 4. Colorectal Cancer

For years, several groups, including our own, have investigated the role of IKKα in colorectal cancer (CRC). Initially, we found that IKKα was aberrantly activated and recruited to the promoter of different Notch target genes such as *hes1*, *hes5*, and *herp2*. Chromatin-bound IKKα constitutively phosphorylates SMRT, leading to its cytoplasmic export and the transcriptional activation of these genes (Figure 1). Conversely, IKKα inhibition, either pharmacologically or by expression of a dominant-negative form of the kinase, restores SMRT chromatin binding, inhibits Notch-dependent gene transcription, and reduces tumor size in a model of CRC xenografts [31]. Similarly, IKKα can phosphorylate the nuclear receptor co-repressor (N-CoR), a nuclear co-repressor homologous to SMRT, thus creating a functional 14-3-3-binding domain and promoting its nuclear export [32]. In a more recent study, we were able to identify the presence of a truncated form of IKKα with a predicted molecular weight of 45 KDa (p45-IKKα) that was specifically activated in the nucleus of CRC cells [33]. This truncated form of IKKα is generated by the proteolytic cleavage of full-length IKKα in the early endosomes by the action of cathepsins. The p45-IKKα form includes the kinase domain, but lacks some regulatory domains at the c-terminal [33]. Nuclear active p45-IKKα forms a complex with full length IKKα and NEMO, and regulates the phosphorylation of SMRT and histone H3. Activated p45-IKKα prevents apoptosis of CRC cells in vitro and it is required for the maintenance of tumor growth in vivo. Consistent with the fact that p45-IKKα is generated in the endosomes, inhibitors of endosome acidification abolish p45-IKKα activation and suppress CRC cell growth both in vitro and in vivo. Moreover, we demonstrated that BRAF activity is required and sufficient to induce p45-IKKα activation, which is TAK1-dependent [34] (Figure 1).

In a different set of experiments, mice deficient in the IKKα kinase activity were protected from intestinal tumor development, which was associated with an enhanced recruitment of interferon γ (IFNγ)-producing M1-like myeloid cells into the tumor. Polarization and accumulation of M1 macrophages in the mutant mice is not cell-autonomous, but depends on the interaction between IKKα-mutant epithelial cells and mutant stromal cells [35].

## 5. Skin Cancer

**Figure 2:**
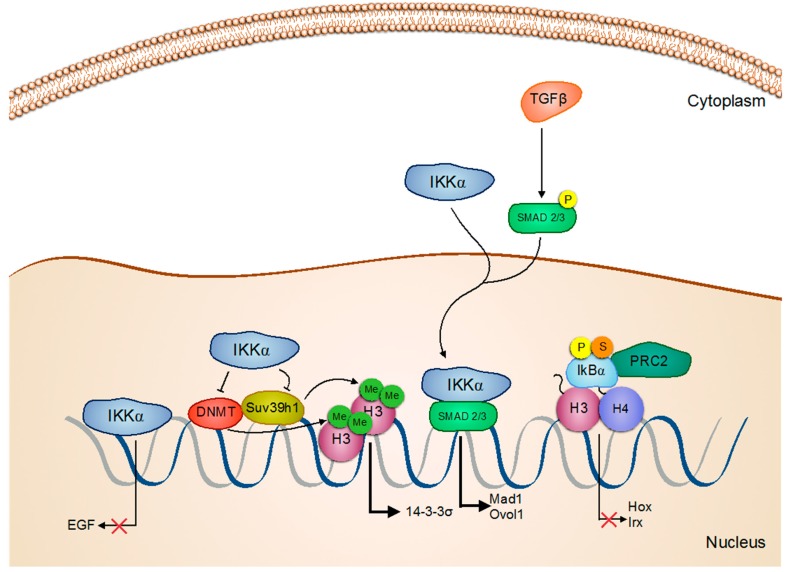
Tumor-suppressing functions of IKKα and IκBα. On one hand, IKKα increases SMAD transcriptional activity and decreases EGF transcription. It also promotes G2/M phase progression by de-repressing 14-3-3σ gene expression through preventing DNA and histone methylation on its promoter. On the other hand, IκBα is bound to histones and nuclear co-repressors, such as PRC2 regulating the expression of genes related to development and differentiation. Arrows: 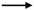 Activation/Regulation/Phosphorylation; 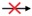 Inactivation; 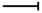 Inhibition

Nuclear IKKα is clearly involved in skin cancer progression, although some controversy exists about its contribution. Whereas different studies have definitively shown that nuclear IKKα in association with SMAD2/3 is required for physiologic skin differentiation [36,37,38], others also indicate that altered IKKα function can directly contribute to specific oncogenic functions. For example, IKKα can bind and repress the promoter of epidermal growth factor (EGF), among others, thus suppressing the EGF receptor/Ras/ERK pathway to prevent squamous cell carcinoma (SCC) [39]. Binding of IKKα to histone H3 at the 14-3-3 sigma locus prevents its hypermethylation by SUV39h1 and supports 14-3-3 sigma expression (Figure 2). Since 14-3-3 sigma controls the cytoplasmic export of the cell cycle-regulatory phosphatase CDC25, the absence of functional IKKα precludes G2/M cell cycle arrest in response to DNA damage, thus contributing to genomic instability and skin cancer [40].

Additional tumor suppressor activity for IKKα in SCC, which is again dependent on its nuclear localization and associated with the transforming growth factor β (TGFβ) pathway, is executed through Myc inhibition [41]. In the same direction, IKKα activates several anti-proliferative Myc antagonists, including Mad1, Mad2, and Ovol1, through Smad2/3, leading to enhanced keratinocyte differentiation [42] (Figure 2). In basal cell carcinoma, *LGR5* expression in also dependent on IKKα and STAT3, suggesting that increased IKKα activity can contribute to oncogenic transformation not only through inflammatory-related signals but also through the regulation of stemness-related genes [43]. In a different study, we found that IKKα induces the chromatin release of phospho-SUMO-IκBα (PS-IκBα), previously identified as a regulator of multiple developmental- and stemness-related genes, such as *HOX* and *IRX*, and its subsequent accumulation in the cytoplasm, which was linked to oncogenic keratinocyte transformation [44] (Figure 1 and Figure 2). The mechanisms by which IKKα promote PS-IκBα inactivation are primarily unknown, but we speculate that nuclear IKKα might phosphorylate PS-IκBα and non-canonical, sites or regulate specific editing enzymes, phosphatases, SUMO-proteases or specific PS-IκBα-interacting proteins.

Recently, it was shown that mice carrying an IKKα variant that specifically localizes in the nucleus of the keratinocytes develop more aggressive tumors in response to chemical carcinogens than control mice. Nuclear IKKα seem to promote tumorigenesis by regulation of *c-myc*, *Maspin*, and *Integrin-α6*, and tumors with nuclear IKKα mimic the characteristics of human skin tumors with a high risk of metastasizing [45]. These results partially overlap our previous findings indicating that nuclear active IKKα plays oncogenic and pro-metastatic roles in SCC, being that its detection is predictive of higher metastatic capacity and worse patient outcome. We also found that nuclear active IKK levels inversely correlated with the levels of the metastasis suppressor Maspin (Figure 1), and tumors negative for this protein were exclusively found in the metastatic group [46].

As mentioned, PS-IκBα was previously detected in fibroblasts [47] and primary keratinocytes [44] as a protein capable of binding the chromatin through the N-terminal tail of histones H2A and H4 [44,47].

Importantly, PS-IκBα also binds histone deacetylases (HDACs) and the polycomb repressive complex 2 (PRC2) to regulate the expression of genes related to development and differentiation in a TNFα-dependent, but NF-κB-independent, manner [44]. Regulation of these genes might contribute to the maintenance of the skin homeostasis, as IκBα-deficient mice die five days after birth due to massive skin inflammation and defective skin differentiation [44,48,49,50]. Supporting a role for nuclear PS-IκBα in skin cancer, nuclear IκBα levels are significantly reduced, or totally lost, in aggressive human SCC and mouse transformed keratinocytes associated with an accumulation of cytoplasmic IκBα and altered *HOX* gene expression (Figure 2). In contrast, IκBα remains nuclear in the normal skin, and also in benign skin lesions, such as elastosis, psoriasis, actinic keratosis, and Bowen disease [44]. Our data might also help to understand previous and unexpected results obtained using a transgenic mouse carrying the non-degradable IκBα mutant, IκBα-SR (for IκBα super repressor) that showed increased and more aggressive tumorigenesis, even in the absence of NF-κB activity [51,52,53,54]. We propose that accumulation of IκBα-SR in the cytoplasm exerts pro-tumorigenic capacities by sequestering PRC2 and HDACs in the cytoplasm leading to inappropriate gene expression of PS-IκBα targets [29,31,32,47] (Figure 1).

## 6. Liver Cancer

Hepatocellular carcinoma (HCC) is one of the most common cancers worldwide and develops frequently in the context of chronic hepatitis, characterized by liver inflammation and hepatocyte apoptosis [55,56]. In this context, the NF-κB pathway can act as a tumor promoter or tumor suppressor [57]. Luedde and colleagues demonstrated that IKKα and IKKβ regulate biliary homeostasis and promote hepatocellular carcinoma by phosphorylating receptor-interacting protein kinase 1 (RIPK1), which is involved in both apoptosis and programmed necrotic cell death (necroptosis), independent of NF-κB. Specifically, loss of IKKα- and IKKβ-dependent RIPK1 phosphorylation in liver parenchymal cells inhibits compensatory proliferation and prevents the development of HCC, but promotes biliary cell paucity and cholestasis [58]. Moreover, IKKβ-depleted hepatocytes display sustained activation of the MKK4/7-JNK signaling cascade, previously identified as a mediator of hepatocellular carcinoma [59]. Deletion of the TAK1 kinase in these same cells induces hepatocyte dysplasia and early carcinogenesis in mice, and this tumor suppressor TAK1 activity is mediated by an NF-κB-independent, but NEMO-dependent, pathway [60].

On the contrary, other studies indicate that NEMO exerts a protective role against HCC through NF-κB-dependent and -independent pathways. In this sense, deletion of NEMO in the liver parenchymal cells (LPC) of 12-month-old mice results in spontaneous hepatocyte apoptosis, which triggers compensatory hepatocyte proliferation, inflammation, activation of liver progenitor cells and, finally, development of chronic hepatitis and HCC [61]. However, ablation of all three NF-κB proteins in LPC able of activating gene transcription (RelA, RelB, and c-Rel) has a limited effect on hepatocyte apoptosis at a young age, indicative of NF-κB-independent activity. Therefore, the canonical NF-κB pathway contributes to the survival of liver cells, but NEMO prevents liver tumorigenesis by NF-κB-independent functions. The mechanism by which NEMO prevents hepatocyte apoptosis is by inhibiting the formation of the death-inducing RIPK1/FADD/caspase-8 signaling complex. Thus, in the absence of NEMO, but high activity of the NF-κB pathway, which induces pro-survival genes, the RIPK1/FADD/caspase-8 complex imposes chronic liver damage, leading to HCC development [61,62,63]. All of these results are clinically relevant since NEMO expression is lost or low in a significant percentage of human HCC correlating with a poor five-year overall survival of patients [64].

## 7. Renal Cancer

Clear cell renal cell carcinomas (ccRCCs) are characterized by the loss of functional von Hippel-Lindau protein (pVHL), which leads to the stabilization of hypoxia-inducible factor alpha (HIFα) and activation of genes related to tumor development and progression, such as chemokine C-X-C motif (CXCR4) [65]. It was found that NEMO stabilizes HIFα via direct interaction and independently of NF-κB signaling. Moreover, NEMO inhibits apoptosis of tumor cells and activates the epithelial-to-mesenchymal transition, thus facilitating the metastatic process [66,67].

## 8. Lung Cancer

In lung cancer, it was shown that IKKα phosphorylates CBP to increase its affinity for NF-κB at the expense of CBP association to p53. Thus, IKKα activity causes increased NF-κB-mediated signaling, but decreased p53-dependent gene expression, leading to cell proliferation and tumor growth. In agreement with this finding, increased CBP phosphorylation and high levels of active IKKα are both detected in human lung tumor tissue compared to the adjacent normal tissue [68].

## 9. Conclusions

As mentioned, NF-κB is a complex and diverse pathway with a clear role in inflammation and immune response. However, there is now increasing evidence that specific elements of the pathway exert NF-κB-independent functions (Table 1), thus increasing the complexity of the NF-κB-related responses. This complexity is even higher in the context of cancer where particular elements could be mutated or aberrantly activated. Most of these functions are due to the accumulation of these members in the nucleus, regulating the expression of onco- or tumor suppressor genes. Here, we have examined some of the non-conventional functions for specific IKK and IκB members that are related to carcinogenesis, which might open new perspectives for future investigations with potential clinical applications.

Among other elements of the pathway, IKKα seem to play a principal role in the regulation, both negatively and positively, of many types of cancer. However, IKKβ and NEMO that are essential components of the canonical IKK complex might also play a role, as it has already been shown in breast, liver, and renal cancer. The recent identification of chromatin-associated PS-IκBα, and its likely regulation by IKKα, add a novel layer of complexity and should lead to the re-evaluation of previous observations and conclusions about the role of IκBα inhibitors in cancer.

In conclusion, a better characterization of these non-canonical functions, how they are accumulated in the nucleus of cancer cells, and how they are integrated or not in the circuits involving NF-κB, should provide a clearer picture of the mechanisms controlling human cancer, thus providing novel elements for therapy assignment.

## Figures and Tables

**Table 1:** Table summarizing the published data on non-conventional functions of the NF-κB members in cancer. The red background shows pro-tumorigenic functions and the green background shows anti-tumorigenic activities. Abbreviations: ERα: estrogen receptor α; SRC-3: nuclear receptor coactivator-3; mTORC: mammalian target of rapamycin complex; SMRT: silencing mediator for retinoid and thyroid receptors; N-CoR: nuclear correpresor; IFNγ: interferon γ; EGF: epidermal growth factor; LGR5: leucine-rich repeat-containing G-protein coupled receptor 5; PS-IκBα: phospho-sumo inhibitor of κBα; EGFR: epidermal growth factor receptor; MMP-9: matric metallopeptidase 9; VEGF-A: vascular endothelial growth factor-A; RIPK1: receptor interacting serine/threonine kinase 1; FOXO3a: forkhead box O3; MKK4/7: mitogen-activated protein kinase kinases 4 and 7; JNK: c-Jun N-terminal kinase; HDAC: histone deacetylase; PRC2: polycomb Repressive Complex 2: NEMO: NFκB essential modulator; NFκB: nuclear factor κB; Casp8: caspase 8; HIFα: hypoxia-inducible factor α; CBP: CREB-binding protein; CRC: colorectal cancer; SCC: squamous cell carcinoma; BCC: basal cell carcinoma; NMSC: non-melanoma skin cancer; HCC: hepatocellular carcinoma; ccRCC: clear cell renal cell carcinoma.

Protein	Substrate	Effect	Cancer Type	References
**IKKα**	Phosphorylation of ERα and SRC-3	Estrogen-dependent gene transcription	Breast Cancer	[20]
	Cooperation with Notch1 to activate transcription of ERα-dependent genes	Cell proliferation	Breast Cancer	[21]
	E2F1 transcription	Cell cycle progression	Breast Cancer	[22]
	Phosphorylation of p27	Expansion of tumour-initiating cells	Breast Cancer	[23]
	Phosphorylation of mTORC	Cell proliferation	Prostate Cancer	[25,26]
	Activation of mTORC2	Akt activation	Prostate Cancer	[27]
	Phosphorylation of SMRT	Increased cell survival Regulation of Notch-dependent gene transcription: Tumour growth	Prostate Cancer CRC	[29] [31]
	Maspin gene repression	Metastasis induction	Prostate Cancer SCC	[30] [46]
	Phosphorylation of NCoR	Increased gene transcription	CRC	[32]
	Regulation of IFNγ-expressing M1-like myeloid cells recruitment	Enhanced tumorigenesis	CRC	[35]
	Repression of EGF transcription	Prevention of SCC	SCC	[39]
	Prevents hypermethylation of 14-3-3sigma through Suv39h1	Maintenance of genomic stability in keratinocytes	Skin Cancer	[40]
	Myc inhibition	Tumour-suppressive activity	SCC	[41]
	Myc inhibition	Keratinocyte proliferation and differentiation	Skin Cancer	[42]
	LGR5 expression	Oncogenic transformation	BCC	[43]
	Chromatin release of PS-IκBα	Oncogenic transformation	Skin Cancer	[44]
	N: c-Myc, Maspin and Integrin-α6 expression: Cyt: Increases EGFR, MMP-9 and VEGF-A activity	Cancer progression	NMSC	[45]
	Phosphorylation of RIPK1	Regulation of cell viability	HCC	[58]
**p45-IKKα**	Phosphorylation of SMRT and Histone H3 Regulation of anti-apoptotic and pro-metastatic genes	Tumour maintenance and apoptosis inhibition Tumour growth and metastasis	CRC CRC	[33] [34]
**IKKβ**	Phosphorylation of FOXO3a	Increased proliferation	Breast Cancer	[24]
	Phosphorylation of RIPK1	Regulation of cell viability	HCC	[58]
	Repression of MKK4/7-JNK signalling cascade	Tumour suppressor	HCC	[59]
**IκBα**	Binding to HDACs and PRC2	Regulation of HOX and IRX: keratinocyte differentiation	SCC	[44]
**TAK1**	Suppression of specific NEMO function	Suppression of procarcinogenic and pronecrotic pathway	HCC	[60]
**NEMO**	NFκB activation	Tumour suppressor	HCC	[61][62]
	Inhibition RIPK1 and Casp8	Suppression of hepatocyte apoptosis	HCC	[62]
	HIFα stabilization	Cell survival	ccRCC	[66]
	Phosphorylation of CBP	Cell proliferation	Lung Cancer	[68]
